# Methyl (3R*,3′S*)-1′,1′′-dimethyl-2,2′′-dioxodispiro­[indoline-3,2′-pyrrolidine-3′,3′′-indoline]-4′-carboxyl­ate

**DOI:** 10.1107/S1600536812037440

**Published:** 2012-09-08

**Authors:** G. Ganesh, Panneer Selvam Yuvaraj, E. Govindan, Boreddy S. R. Reddy, A. SubbiahPandi

**Affiliations:** a Department of Physics, S.M.K. Fomra Institute of Technology, Thaiyur, Chennai 603 103, India; b Industrial Chemistry Laboratory, Central Leather Research Institute, Adyar, Chennai 600 020, India; c Department of Physics, Presidency College (Autonomous), Chennai 600 005, India

## Abstract

In the title compound, C_22_H_21_N_3_O_4_, the central pyrrolidine ring adopts an envelope conformation with the N atom in the flap position. The indoline ring systems are almost perpendic­ular to the mean plane of the pyrrolidine ring, making dihedral angles of 86.4 (8) and 83.1 (8)°. The acetate group attached to the pyrrolidine ring assumes an extended conformation. In thecrystal, N—H⋯O hydrogen bonds result in the formation of a *C*(7) chain running along [100]. The crystal packing also features π–π inter­actions [centroid–centroid distance = 3.2032 (11) Å].

## Related literature

For the biological activity of spiro-pyrrolidine derivatives, see: Obniska *et al.* (2003 ▶); Peddi *et al.* (2004 ▶); Kaminski & Obniska (2008 ▶); Stylianakis *et al.* (2003 ▶); Waldmann (1995 ▶); Suzuki *et al.* (1994 ▶); Huryn *et al.* (1991 ▶). For a related structure, see: Wei *et al.* (2011 ▶). For puckering parameters, see: Cremer & Pople (1975 ▶). For asymmetry parameters, see: Nardelli (1983 ▶).
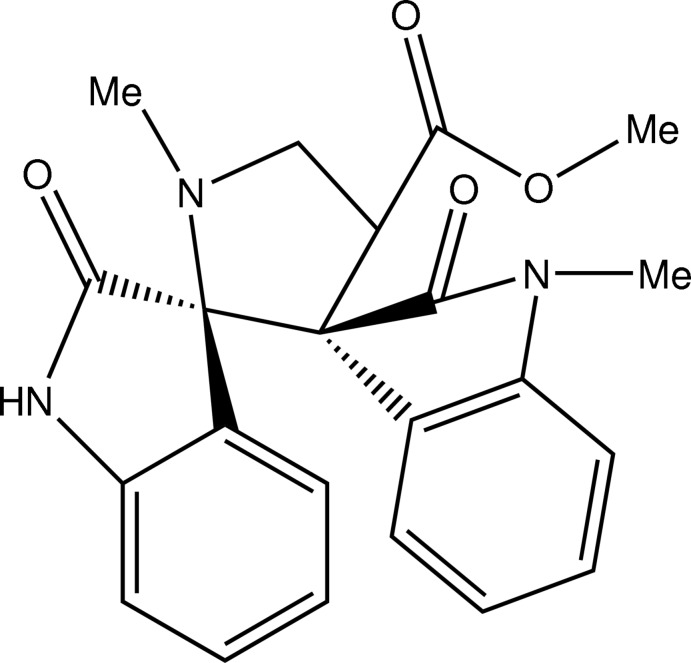


## Experimental

### 

#### Crystal data

C_22_H_21_N_3_O_4_
*M* *_r_* = 391.42
Monoclinic, 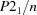

*a* = 9.8244 (4) Å
*b* = 12.7193 (5) Å
*c* = 15.7630 (6) Å
β = 95.474 (2)°
*V* = 1960.75 (13) Å^3^
*Z* = 4
Mo *K*α radiation
μ = 0.09 mm^−1^
*T* = 293 K
0.25 × 0.22 × 0.19 mm

#### Data collection

Bruker APEXII CCD area-detector diffractometer
Absorption correction: multi-scan (*SADABS*; Sheldrick, 1996 ▶) *T*_min_ = 0.978, *T*_max_ = 0.983
17984 measured reflections
3691 independent reflections
2716 reflections with *I* > 2σ(*I*)
*R*_int_ = 0.034

#### Refinement

*R*[*F* ^2^ > 2σ(*F* ^2^)] = 0.043
*wR*(*F* ^2^) = 0.110
*S* = 1.01
3691 reflections
265 parameters
H-atom parameters constrained
Δρ_max_ = 0.21 e Å^−3^
Δρ_min_ = −0.18 e Å^−3^

### 

Data collection: *APEX2* (Bruker, 2008 ▶); cell refinement: *SAINT* (Bruker, 2008 ▶); data reduction: *SAINT*; program(s) used to solve structure: *SHELXS97* (Sheldrick, 2008 ▶); program(s) used to refine structure: *SHELXL97* (Sheldrick, 2008 ▶); molecular graphics: *ORTEP-3* (Farrugia, 1997 ▶); software used to prepare material for publication: *SHELXL97* and *PLATON* (Spek, 2009 ▶).

## Supplementary Material

Crystal structure: contains datablock(s) global, I. DOI: 10.1107/S1600536812037440/bx2423sup1.cif

Structure factors: contains datablock(s) I. DOI: 10.1107/S1600536812037440/bx2423Isup2.hkl

Additional supplementary materials:  crystallographic information; 3D view; checkCIF report

## Figures and Tables

**Table 1:** Hydrogen-bond geometry (Å, °)

*D*—H⋯*A*	*D*—H	H⋯*A*	*D*⋯*A*	*D*—H⋯*A*
N2—H2*A*⋯O4^i^	0.86	2.08	2.8935 (19)	157

Symmetry code: (i) 
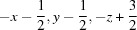
.

**Fig. 1.**
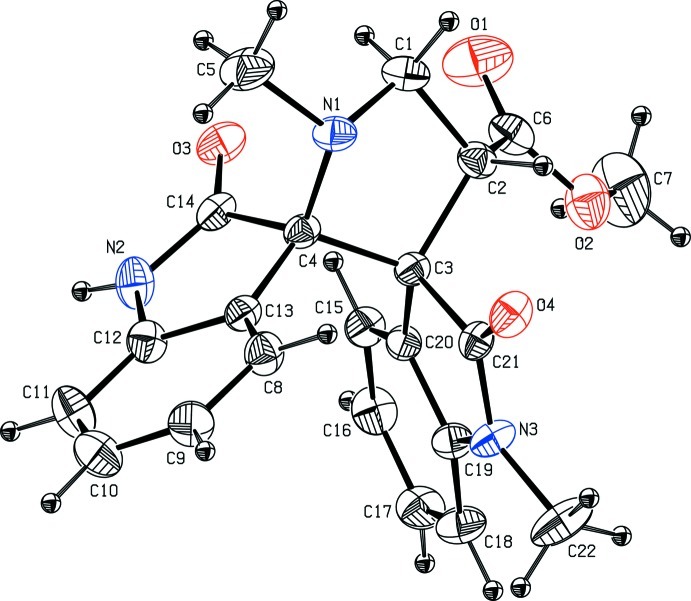
The structure of showing the atom-numbering scheme. The displacement ellipsoids are drawn at the 30% probability level.

**Fig. 2.**
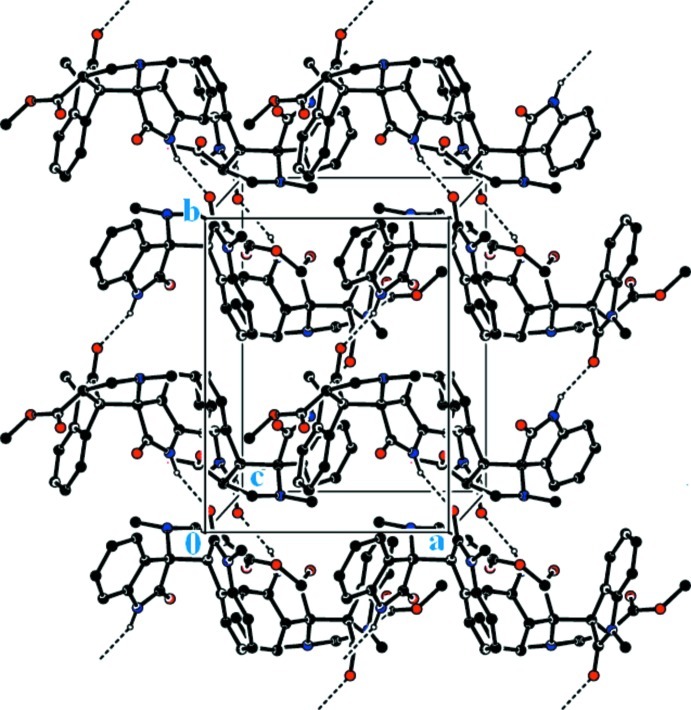
The molecular packing viewed down the c axis. Dashed lines shows the intermolecular N—H···O hydrogen bonds.

## supplementary crystallographic information

### Comment

Spiro-pyrrolidine derivatives are unique tetracyclic 5-HT(2 A) receptor antagonist (Obniska *et al.*, 2003; Peddi *et al.*, 2004). These derivatives possess anticonvulsant (Kaminski & Obniska, 2008) and anti-influenza virus (Stylianakis *et al.*, 2003) activities. Highly functionalized pyrrolidines have gained much interest in the past few years as they constitute the main structural element of many natural and synthetic pharmacologically active compounds (Waldmann, 1995). Optically active pyrrolidines have been used as intermediates, chiral ligands or auxiliaries in controlled asymmetric synthesis (Suzuki *et al.*, 1994; Huryn *et al.*, 1991). In view of these importance and continuation of our work on the crystal structure analyis of spiro-pyrrolidine derivatives, the crystal structure of the title compound has been carried out and the results are presented here.

X-Ray analysis confirms the molecular structure and atom connectivity as illustrated in Fig. 1. The geometry of pyrrolidine and indoline group systems are comparable with the related structure (Wei *et al.*, 2011). The sum of the angles at N1 [336.2 (1)°] and N3 [358.8 (1)°] of the pyrrolidine rings are in accordance with *sp*^3^ and *sp*^2^ hybridizations. The indoline ring systems [N2/C4/C8—C14 and N3/C3/C15—C21] makes the dihedral angles of 86.4 (8) ° and 83.1 (8)° with respect to the mean plane of the pyrrolidine ring system, it clearly shows the indoline rings attached to the pyrrolidine ring system are almost perpendicular to each other. The acetate group assumes an extended conformation as can be seen from torsion angle C2—C6—O2—C7 = 175.1 (2) °.

The pyrrolidine rings [N1/C1—C4] adopts envelope conformation, with the puckering parameters q_2_ and φ (Cremer & Pople, 1975) and the smallest displacement asymmetric parameters, Δ, (Nardelli *et al.*, 1983) as follows: q_2_ = 0.4044 (2) Å, φ = 354.1 (3)° and Δ_s_(N1) = 4.22 (2)°. In the crystal the molecules are linked by intermolecular N2—H2A···O4 (-1/2 - *x*,-1/2 + *y*,3/2 - *z*) hydrogen bonds result in the formation of infinite C(7) chain running along *a* axis. The crystal packing is further stabilized by π–π stacking interaction between *Cg*2 and *Cg*3 rings at *x*,*y*,*z*. The centroid–centroid distance between these two rings is 3.2032 (11) Å]. *Cg*2 and *Cg*3 are the centroid of the N2/C4/C12/C13/C14 and N3/C3/C19/C20/C21 rings.

### Experimental

To a mixture of 1eq of (*E*)-methyl 2-(1-methyl-2-oxoindolin-3-ylidene) acetate, 1eq of isatin and 1.5eq of sarcosine were dissolved in acetonitrile. This rection mixture refluxed at 80°C for 8 h. Completion of reaction monitor by thin layer chromatography. The reaction mixture was extracted with ethyl acetate and water. The product was dried and purified by column chromatography using ethyl acetate and hexane (1:9) as an elutent to afford pure Dispiro oxindole. (Yield:90%). Single crystals suitable for X-ray diffraction were obtained by slow evaporation of a solution of the title compound in ethyl acetate at room temperature.

### Refinement

All H atoms were fixed geometrically and allowed to ride on their parent C atoms, with C—H distances fixed in the range 0.93–0.97 Å with *U*_iso_(H) = 1.5*U*_eq_(C) for methyl H 1.2*U*_eq_(C) for other H atoms.

### Crystal data

**Table:**

C_22_H_21_N_3_O_4_	*F*(000) = 824
*M**_r_* = 391.42	*D*_x_ = 1.326 Mg m^−^^3^
Monoclinic, *P*2_1_/*n*	Mo *K*α radiation, λ = 0.71073 Å
Hall symbol: -P 2yn	Cell parameters from 3691 reflections
*a* = 9.8244 (4) Å	θ = 2.1–25.7°
*b* = 12.7193 (5) Å	µ = 0.09 mm^−^^1^
*c* = 15.7630 (6) Å	*T* = 293 K
β = 95.474 (2)°	Block, colourless
*V* = 1960.75 (13) Å^3^	0.25 × 0.22 × 0.19 mm
*Z* = 4	

### Data collection

**Table:**

Bruker APEXII CCD area-detector diffractometer	3691 independent reflections
Radiation source: fine-focus sealed tube	2716 reflections with *I* > 2σ(*I*)
Graphite monochromator	*R*_int_ = 0.034
ω and φ scans	θ_max_ = 25.7°, θ_min_ = 2.1°
Absorption correction: multi-scan (*SADABS*; Sheldrick, 1996)	*h* = −11→11
*T*_min_ = 0.978, *T*_max_ = 0.983	*k* = −15→15
17984 measured reflections	*l* = −19→15

### Refinement

**Table:**

Refinement on *F*^2^	Primary atom site location: structure-invariant direct methods
Least-squares matrix: full	Secondary atom site location: difference Fourier map
*R*[*F*^2^ > 2σ(*F*^2^)] = 0.043	Hydrogen site location: inferred from neighbouring sites
*wR*(*F*^2^) = 0.110	H-atom parameters constrained
*S* = 1.01	*w* = 1/[σ^2^(*F*_o_^2^) + (0.0457*P*)^2^ + 0.6361*P*] where *P* = (*F*_o_^2^ + 2*F*_c_^2^)/3
3691 reflections	(Δ/σ)_max_ < 0.001
265 parameters	Δρ_max_ = 0.21 e Å^−^^3^
0 restraints	Δρ_min_ = −0.18 e Å^−^^3^

### Special details

**Table:**

Geometry. All e.s.d.'s (except the e.s.d. in the dihedral angle between two l.s. planes) are estimated using the full covariance matrix. The cell e.s.d.'s are taken into account individually in the estimation of e.s.d.'s in distances, angles and torsion angles; correlations between e.s.d.'s in cell parameters are only used when they are defined by crystal symmetry. An approximate (isotropic) treatment of cell e.s.d.'s is used for estimating e.s.d.'s involving l.s. planes.
Refinement. Refinement of *F*^2^ against ALL reflections. The weighted *R*-factor *wR* and goodness of fit *S* are based on *F*^2^, conventional *R*-factors *R* are based on *F*, with *F* set to zero for negative *F*^2^. The threshold expression of *F*^2^ > σ(*F*^2^) is used only for calculating *R*-factors(gt) *etc*. and is not relevant to the choice of reflections for refinement. *R*-factors based on *F*^2^ are statistically about twice as large as those based on *F*, and *R*- factors based on ALL data will be even larger.

### Fractional atomic coordinates and isotropic or equivalent isotropic displacement parameters (Å^2^)

**Table:**

	*x*	*y*	*z*	*U*_iso_*/*U*_eq_	
C1	−0.1007 (2)	−0.03758 (15)	0.60269 (11)	0.0486 (5)	
H1A	−0.0871	0.0299	0.5761	0.058*	
H1B	−0.1229	−0.0896	0.5586	0.058*	
C2	0.02541 (18)	−0.06977 (13)	0.65991 (11)	0.0382 (4)	
H2	0.0738	−0.0054	0.6791	0.046*	
C3	−0.03026 (17)	−0.12230 (12)	0.74001 (10)	0.0320 (4)	
C4	−0.18972 (17)	−0.12132 (12)	0.71699 (10)	0.0335 (4)	
C5	−0.3439 (2)	−0.01745 (19)	0.61685 (14)	0.0640 (6)	
H5A	−0.3650	−0.0755	0.5789	0.096*	
H5B	−0.3472	0.0468	0.5848	0.096*	
H5C	−0.4096	−0.0146	0.6582	0.096*	
C6	0.1228 (2)	−0.13630 (15)	0.61579 (13)	0.0488 (5)	
C7	0.3381 (3)	−0.2173 (2)	0.6299 (2)	0.1044 (10)	
H7A	0.3031	−0.2878	0.6288	0.157*	
H7B	0.4230	−0.2146	0.6654	0.157*	
H7C	0.3531	−0.1961	0.5730	0.157*	
C8	−0.30396 (19)	−0.03885 (14)	0.84650 (11)	0.0446 (5)	
H8	−0.2595	0.0255	0.8446	0.053*	
C9	−0.3969 (2)	−0.05603 (16)	0.90536 (12)	0.0519 (5)	
H9	−0.4152	−0.0026	0.9429	0.062*	
C10	−0.4627 (2)	−0.15059 (17)	0.90921 (13)	0.0588 (6)	
H10	−0.5229	−0.1614	0.9504	0.071*	
C11	−0.4404 (2)	−0.23012 (16)	0.85248 (14)	0.0596 (6)	
H11	−0.4859	−0.2941	0.8540	0.071*	
C12	−0.34875 (19)	−0.21128 (13)	0.79372 (12)	0.0425 (4)	
C13	−0.27769 (17)	−0.11765 (12)	0.79085 (10)	0.0348 (4)	
C14	−0.23636 (18)	−0.22843 (13)	0.67501 (11)	0.0388 (4)	
C15	0.04532 (19)	−0.32158 (13)	0.73222 (12)	0.0412 (4)	
H15	0.0074	−0.3327	0.6766	0.049*	
C16	0.1163 (2)	−0.40147 (14)	0.77744 (13)	0.0497 (5)	
H16	0.1263	−0.4664	0.7516	0.060*	
C17	0.1718 (2)	−0.38606 (15)	0.85940 (13)	0.0531 (5)	
H17	0.2176	−0.4411	0.8887	0.064*	
C18	0.1611 (2)	−0.29021 (15)	0.89939 (13)	0.0509 (5)	
H18	0.1992	−0.2793	0.9550	0.061*	
C19	0.09178 (18)	−0.21159 (13)	0.85362 (11)	0.0381 (4)	
C20	0.03200 (17)	−0.22564 (12)	0.77117 (10)	0.0330 (4)	
C21	0.00958 (17)	−0.04954 (13)	0.81677 (11)	0.0353 (4)	
C22	0.1426 (3)	−0.06320 (19)	0.95731 (14)	0.0729 (7)	
H22A	0.1281	0.0114	0.9576	0.109*	
H22B	0.2389	−0.0775	0.9601	0.109*	
H22C	0.1056	−0.0941	1.0057	0.109*	
N1	−0.20775 (16)	−0.03125 (11)	0.66017 (9)	0.0420 (4)	
N2	−0.31930 (16)	−0.27579 (12)	0.72636 (10)	0.0502 (4)	
H2A	−0.3505	−0.3386	0.7185	0.060*	
N3	0.07510 (16)	−0.10775 (11)	0.87955 (9)	0.0424 (4)	
O1	0.1005 (2)	−0.17271 (15)	0.54647 (10)	0.0875 (6)	
O2	0.24061 (15)	−0.14729 (11)	0.66334 (10)	0.0639 (4)	
O3	−0.20458 (14)	−0.26255 (10)	0.60748 (8)	0.0524 (4)	
O4	−0.00852 (13)	0.04480 (9)	0.82033 (8)	0.0467 (3)	

### Atomic displacement parameters (Å^2^)

**Table:**

	*U*^11^	*U*^22^	*U*^33^	*U*^12^	*U*^13^	*U*^23^
C1	0.0598 (13)	0.0488 (11)	0.0373 (10)	0.0048 (9)	0.0044 (9)	0.0115 (8)
C2	0.0463 (11)	0.0311 (9)	0.0376 (10)	−0.0019 (7)	0.0053 (8)	0.0030 (7)
C3	0.0364 (9)	0.0272 (8)	0.0316 (9)	0.0002 (7)	−0.0011 (7)	0.0005 (7)
C4	0.0381 (10)	0.0287 (8)	0.0322 (9)	0.0003 (7)	−0.0030 (7)	0.0000 (7)
C5	0.0575 (14)	0.0739 (15)	0.0581 (13)	0.0212 (11)	−0.0077 (11)	0.0133 (11)
C6	0.0589 (14)	0.0418 (10)	0.0472 (12)	−0.0001 (9)	0.0133 (10)	0.0041 (9)
C7	0.0647 (17)	0.100 (2)	0.153 (3)	0.0295 (16)	0.0350 (19)	0.003 (2)
C8	0.0471 (11)	0.0401 (10)	0.0467 (11)	−0.0054 (8)	0.0052 (9)	−0.0079 (8)
C9	0.0553 (13)	0.0555 (12)	0.0463 (11)	0.0004 (10)	0.0114 (10)	−0.0108 (9)
C10	0.0611 (14)	0.0611 (13)	0.0573 (13)	0.0019 (11)	0.0223 (11)	0.0040 (10)
C11	0.0616 (14)	0.0442 (11)	0.0761 (15)	−0.0083 (10)	0.0230 (12)	0.0033 (10)
C12	0.0453 (11)	0.0330 (9)	0.0490 (11)	0.0002 (8)	0.0039 (9)	−0.0017 (8)
C13	0.0363 (9)	0.0324 (8)	0.0347 (9)	0.0006 (7)	−0.0022 (7)	−0.0002 (7)
C14	0.0396 (10)	0.0368 (9)	0.0377 (10)	0.0023 (8)	−0.0083 (8)	−0.0044 (8)
C15	0.0454 (11)	0.0338 (9)	0.0438 (10)	0.0007 (8)	0.0014 (8)	−0.0018 (8)
C16	0.0527 (12)	0.0321 (9)	0.0648 (13)	0.0088 (8)	0.0088 (10)	0.0004 (9)
C17	0.0518 (12)	0.0457 (11)	0.0610 (13)	0.0157 (9)	0.0019 (10)	0.0157 (10)
C18	0.0537 (12)	0.0548 (12)	0.0426 (11)	0.0146 (10)	−0.0042 (9)	0.0074 (9)
C19	0.0402 (10)	0.0384 (9)	0.0354 (10)	0.0053 (8)	0.0016 (8)	0.0014 (7)
C20	0.0335 (9)	0.0306 (8)	0.0345 (9)	0.0005 (7)	0.0019 (7)	0.0016 (7)
C21	0.0337 (9)	0.0328 (9)	0.0387 (10)	−0.0011 (7)	−0.0009 (8)	−0.0043 (7)
C22	0.0833 (17)	0.0762 (15)	0.0525 (13)	0.0234 (13)	−0.0287 (12)	−0.0264 (11)
N1	0.0463 (9)	0.0408 (8)	0.0379 (8)	0.0079 (7)	−0.0011 (7)	0.0096 (6)
N2	0.0509 (10)	0.0338 (8)	0.0666 (11)	−0.0094 (7)	0.0093 (8)	−0.0138 (7)
N3	0.0491 (9)	0.0421 (8)	0.0334 (8)	0.0079 (7)	−0.0091 (7)	−0.0070 (6)
O1	0.1076 (15)	0.1034 (14)	0.0525 (10)	0.0297 (11)	0.0128 (9)	−0.0193 (9)
O2	0.0449 (9)	0.0626 (9)	0.0854 (11)	0.0047 (7)	0.0118 (8)	0.0021 (8)
O3	0.0633 (9)	0.0526 (8)	0.0396 (7)	0.0027 (7)	−0.0040 (6)	−0.0150 (6)
O4	0.0511 (8)	0.0309 (7)	0.0558 (8)	0.0023 (5)	−0.0061 (6)	−0.0089 (6)

### Geometric parameters (Å, º)

**Table:**

C1—N1	1.455 (2)	C9—H9	0.9300
C1—C2	1.517 (3)	C10—C11	1.381 (3)
C1—H1A	0.9700	C10—H10	0.9300
C1—H1B	0.9700	C11—C12	1.373 (3)
C2—C6	1.498 (3)	C11—H11	0.9300
C2—C3	1.572 (2)	C12—C13	1.383 (2)
C2—H2	0.9800	C12—N2	1.394 (2)
C3—C20	1.512 (2)	C14—O3	1.217 (2)
C3—C21	1.544 (2)	C14—N2	1.345 (2)
C3—C4	1.574 (2)	C15—C20	1.378 (2)
C4—N1	1.454 (2)	C15—C16	1.390 (2)
C4—C13	1.516 (2)	C15—H15	0.9300
C4—C14	1.564 (2)	C16—C17	1.368 (3)
C5—N1	1.454 (2)	C16—H16	0.9300
C5—H5A	0.9600	C17—C18	1.381 (3)
C5—H5B	0.9600	C17—H17	0.9300
C5—H5C	0.9600	C18—C19	1.375 (2)
C6—O1	1.188 (2)	C18—H18	0.9300
C6—O2	1.325 (2)	C19—C20	1.386 (2)
C7—O2	1.444 (3)	C19—N3	1.397 (2)
C7—H7A	0.9600	C21—O4	1.2151 (19)
C7—H7B	0.9600	C21—N3	1.349 (2)
C7—H7C	0.9600	C22—N3	1.452 (2)
C8—C13	1.372 (2)	C22—H22A	0.9600
C8—C9	1.380 (3)	C22—H22B	0.9600
C8—H8	0.9300	C22—H22C	0.9600
C9—C10	1.369 (3)	N2—H2A	0.8600
			
N1—C1—C2	104.04 (13)	C12—C11—H11	121.2
N1—C1—H1A	110.9	C10—C11—H11	121.2
C2—C1—H1A	110.9	C11—C12—C13	122.53 (17)
N1—C1—H1B	110.9	C11—C12—N2	127.46 (17)
C2—C1—H1B	110.9	C13—C12—N2	109.86 (16)
H1A—C1—H1B	109.0	C8—C13—C12	118.78 (16)
C6—C2—C1	113.46 (15)	C8—C13—C4	132.01 (15)
C6—C2—C3	114.81 (14)	C12—C13—C4	108.98 (14)
C1—C2—C3	105.38 (14)	O3—C14—N2	125.97 (16)
C6—C2—H2	107.6	O3—C14—C4	126.24 (16)
C1—C2—H2	107.6	N2—C14—C4	107.79 (14)
C3—C2—H2	107.6	C20—C15—C16	118.90 (17)
C20—C3—C21	101.66 (13)	C20—C15—H15	120.6
C20—C3—C2	118.00 (14)	C16—C15—H15	120.6
C21—C3—C2	107.05 (13)	C17—C16—C15	121.00 (17)
C20—C3—C4	116.40 (13)	C17—C16—H16	119.5
C21—C3—C4	110.38 (13)	C15—C16—H16	119.5
C2—C3—C4	103.08 (13)	C16—C17—C18	121.11 (17)
N1—C4—C13	113.82 (13)	C16—C17—H17	119.4
N1—C4—C14	114.33 (13)	C18—C17—H17	119.4
C13—C4—C14	100.74 (13)	C19—C18—C17	117.26 (18)
N1—C4—C3	102.03 (13)	C19—C18—H18	121.4
C13—C4—C3	116.81 (13)	C17—C18—H18	121.4
C14—C4—C3	109.61 (13)	C18—C19—C20	122.87 (16)
N1—C5—H5A	109.5	C18—C19—N3	126.89 (17)
N1—C5—H5B	109.5	C20—C19—N3	110.20 (14)
H5A—C5—H5B	109.5	C15—C20—C19	118.84 (15)
N1—C5—H5C	109.5	C15—C20—C3	132.68 (16)
H5A—C5—H5C	109.5	C19—C20—C3	108.35 (14)
H5B—C5—H5C	109.5	O4—C21—N3	124.74 (15)
O1—C6—O2	123.6 (2)	O4—C21—C3	126.93 (15)
O1—C6—C2	125.3 (2)	N3—C21—C3	108.27 (13)
O2—C6—C2	111.05 (17)	N3—C22—H22A	109.5
O2—C7—H7A	109.5	N3—C22—H22B	109.5
O2—C7—H7B	109.5	H22A—C22—H22B	109.5
H7A—C7—H7B	109.5	N3—C22—H22C	109.5
O2—C7—H7C	109.5	H22A—C22—H22C	109.5
H7A—C7—H7C	109.5	H22B—C22—H22C	109.5
H7B—C7—H7C	109.5	C5—N1—C4	115.91 (15)
C13—C8—C9	119.40 (17)	C5—N1—C1	113.71 (15)
C13—C8—H8	120.3	C4—N1—C1	106.69 (13)
C9—C8—H8	120.3	C14—N2—C12	112.12 (15)
C10—C9—C8	120.93 (18)	C14—N2—H2A	123.9
C10—C9—H9	119.5	C12—N2—H2A	123.9
C8—C9—H9	119.5	C21—N3—C19	111.43 (14)
C9—C10—C11	120.68 (19)	C21—N3—C22	123.54 (16)
C9—C10—H10	119.7	C19—N3—C22	123.99 (15)
C11—C10—H10	119.7	C6—O2—C7	115.6 (2)
C12—C11—C10	117.60 (18)		
			
N1—C1—C2—C6	148.17 (15)	C15—C16—C17—C18	−1.1 (3)
N1—C1—C2—C3	21.75 (17)	C16—C17—C18—C19	0.4 (3)
C6—C2—C3—C20	7.8 (2)	C17—C18—C19—C20	1.0 (3)
C1—C2—C3—C20	133.43 (15)	C17—C18—C19—N3	−176.25 (18)
C6—C2—C3—C21	121.57 (16)	C16—C15—C20—C19	1.0 (3)
C1—C2—C3—C21	−112.84 (15)	C16—C15—C20—C3	176.17 (17)
C6—C2—C3—C4	−122.00 (16)	C18—C19—C20—C15	−1.7 (3)
C1—C2—C3—C4	3.59 (16)	N3—C19—C20—C15	175.92 (15)
C20—C3—C4—N1	−158.29 (13)	C18—C19—C20—C3	−177.98 (17)
C21—C3—C4—N1	86.55 (14)	N3—C19—C20—C3	−0.31 (19)
C2—C3—C4—N1	−27.49 (15)	C21—C3—C20—C15	−173.52 (18)
C20—C3—C4—C13	76.93 (18)	C2—C3—C20—C15	−56.9 (3)
C21—C3—C4—C13	−38.23 (18)	C4—C3—C20—C15	66.5 (2)
C2—C3—C4—C13	−152.27 (13)	C21—C3—C20—C19	2.00 (17)
C20—C3—C4—C14	−36.75 (19)	C2—C3—C20—C19	118.66 (16)
C21—C3—C4—C14	−151.91 (13)	C4—C3—C20—C19	−117.97 (15)
C2—C3—C4—C14	94.05 (14)	C20—C3—C21—O4	174.00 (17)
C1—C2—C6—O1	−8.8 (3)	C2—C3—C21—O4	49.6 (2)
C3—C2—C6—O1	112.5 (2)	C4—C3—C21—O4	−61.9 (2)
C1—C2—C6—O2	169.32 (15)	C20—C3—C21—N3	−3.08 (17)
C3—C2—C6—O2	−69.4 (2)	C2—C3—C21—N3	−127.46 (15)
C13—C8—C9—C10	0.3 (3)	C4—C3—C21—N3	121.04 (15)
C8—C9—C10—C11	−2.0 (3)	C13—C4—N1—C5	−62.0 (2)
C9—C10—C11—C12	1.2 (3)	C14—C4—N1—C5	53.0 (2)
C10—C11—C12—C13	1.2 (3)	C3—C4—N1—C5	171.25 (15)
C10—C11—C12—N2	−174.0 (2)	C13—C4—N1—C1	170.24 (14)
C9—C8—C13—C12	2.0 (3)	C14—C4—N1—C1	−74.73 (18)
C9—C8—C13—C4	175.81 (18)	C3—C4—N1—C1	43.49 (16)
C11—C12—C13—C8	−2.8 (3)	C2—C1—N1—C5	−170.83 (16)
N2—C12—C13—C8	173.15 (16)	C2—C1—N1—C4	−41.80 (18)
C11—C12—C13—C4	−177.96 (18)	O3—C14—N2—C12	−172.78 (17)
N2—C12—C13—C4	−2.0 (2)	C4—C14—N2—C12	6.5 (2)
N1—C4—C13—C8	−46.1 (3)	C11—C12—N2—C14	172.67 (19)
C14—C4—C13—C8	−168.94 (19)	C13—C12—N2—C14	−3.0 (2)
C3—C4—C13—C8	72.5 (2)	O4—C21—N3—C19	−174.02 (17)
N1—C4—C13—C12	128.17 (16)	C3—C21—N3—C19	3.14 (19)
C14—C4—C13—C12	5.34 (17)	O4—C21—N3—C22	−5.3 (3)
C3—C4—C13—C12	−113.25 (16)	C3—C21—N3—C22	171.91 (18)
N1—C4—C14—O3	49.8 (2)	C18—C19—N3—C21	175.70 (18)
C13—C4—C14—O3	172.23 (17)	C20—C19—N3—C21	−1.8 (2)
C3—C4—C14—O3	−64.1 (2)	C18—C19—N3—C22	7.0 (3)
N1—C4—C14—N2	−129.55 (15)	C20—C19—N3—C22	−170.56 (19)
C13—C4—C14—N2	−7.07 (17)	O1—C6—O2—C7	−6.8 (3)
C3—C4—C14—N2	116.63 (15)	C2—C6—O2—C7	175.05 (19)
C20—C15—C16—C17	0.3 (3)		

### Hydrogen-bond geometry (Å, º)

**Table:**

*D*—H···*A*	*D*—H	H···*A*	*D*···*A*	*D*—H···*A*
N2—H2*A*···O4^i^	0.86	2.08	2.8935 (19)	157

Symmetry code: (i) −*x*−1/2, *y*−1/2, −*z*+3/2.
